# Complementary feeding practices and associated factors among HIV positive mothers in Southern Ethiopia

**DOI:** 10.1186/s41043-015-0006-0

**Published:** 2015-05-01

**Authors:** Demewoz Haile, Tefera Belachew, Getenesh Berhanu, Tesfaye Setegn, Sibhatu Biadgilign

**Affiliations:** 1 Department of Public Health, College of Medicine and Health Sciences, Madawalabu University, Madawalabu, Ethiopia; 2 Population and Family Health Department, College of Public Health and Medical Sciences Jimma University, Jimma, Ethiopia; 3 Department of Applied Human Nutrition, School of Food Sciences and Nutrition, Hawassa University, Hawassa, Ethiopia; 4 Department of Reproductive Health, College of Medicine and Health Sciences, Bahir Dar University, Bahir Dar, Ethiopia; 5 Independent Public Health Research Consultants, Addis Ababa, Ethiopia

## Abstract

The objective of this study was to assess complementary feeding practices and associated factors among HIV exposed infants in Sidama Zone, Southern Ethiopia. An institutional based cross-sectional study with cluster random sampling technique was employed and all HIV exposed infants aged 6-17 months found in randomly selected health institutions in Sidama zone, Southern Ethiopia were included. A 24-hour dietary recall and 7-day quasi-food group frequency was used to assess complementary feeding practices. The prevalence of timely initiation of complementary feeding (6-8 months) was 42% [95% CI: (30-54%)]. Of all the HIV exposed infants aged 6-17 months, 40.7% had practiced bottle-feeding. About 65.6% and 53.3% of HIV exposed infants did not receive the recommended number of food groups and frequency of complementary feeding in the last 24 hours respectively. Pulse (plant protein) was consumed by only 22.5% of the infants while only 9.9% of the infants consumed animal source food in the last 24 hours. Presence of infant food prohibition (β = -0.342, P = 0.001) and age of the infant (β = 0.311, P = 0.001) were found to be an independent predictors of dietary diversity. Presence of infant food prohibition (β = -0.181, P = 0.02) and age of infant (β = 0.388, P < 0.001) were also the predictors of 24 hour meal frequency. Having lower educational status [AOR = (0.21, 95% CI (0.062-0.71)] was an independent negative predictor of bottle-feeding practice. Many of the complementary feeding practices like meal frequency; dietary diversity and bottle-feeding were sub-optimal. Nutrition education should be designed for improving complementary feeding practices of HIV exposed infants in Sidama Zone, Southern Ethiopia. Mothers with higher educational status should be also targeted for nutrition education especially on bottle feeding practice.

## Background

Complementary feeding is an effective child survival strategy and is ranked among the top life-saving interventions for children under 5 years and can prevent about 6% of under-five mortality [1]. For a child’s healthy and productive development adequate quality and quantity complementary food should be started at 6 months and increased appropriately as required [2].

The 2010 WHO infant feeding and HIV guideline recommends starting adequate complementary foods and continuing to breastfeed till the first 12 months of life for HIV exposed infants. The duration of breastfeeding in HIV positive mothers is short as compared to the general population [3]. This would make mothers to demand complementary food in order to increase the HIV free survival rates of their infant, which affects the nutritional and health status of children negatively. In this regard, HIV infected mothers may have an over emphasis on complementary feeding to avoid HIV infection of their infant. This over emphasis of mothers on complementary feeding practice will lead infants to develop malnutrition and diarrhea. [3-7] On the other hand, HIV infection associated with higher risk of household level food insecurity affects the nutritional status of children who need complementary food [6,7].

Infants and young children are at increased risk of malnutrition starting from six months, when breast milk alone is no longer sufficient to meet all the nutritional requirements of infants [8]. In most developing countries, children are introduced directly to the regular household diet made of cereal or starchy root crops which is the major cause for the high incidence of child malnutrition, morbidity and mortality [9-11]. Malnutrition is responsible for over half of all childhood deaths [12] and it increased significantly in HIV exposed infants after 6 months of age which could be partly due to inappropriate feeding practices [13,14]. HIV positive children have significantly poorer nutritional outcomes than their HIV negative counterparts [15]. Both HIV exposure and HIV infection exacerbate childhood malnutrition. Children living with HIV or born into HIV positive mothers are a high-risk group with special needs [16]. Poor complementary feeding practices coupled with high rates of infectious diseases are the principal proximate causes of malnutrition during the first two years of life. For this reason, it is essential to ensure that caregivers are provided with appropriate guidance on optimal infant and young child feeding practices [17]. However, in Ethiopia, there is a paucity of evidence on complementary feeding practices of HIV exposed children. Therefore, the objective of this study is to assess the complementary feeding practices and associated factors among HIV exposed children in Sidama Zone, Southern Ethiopia.

## Methods

### Study setting and sample

A facility based cross sectional study was conducted in 10 randomly selected government health institutions (3 hospitals and 7 health centers) providing ART and PMTCT services in Sidama Zone, Southern Ethiopia between February and April 2012. Sidama zone is one of the fourteen zones in the Southern Nations and Nationalities regional state. According to the recent census in 2007, 50.48% of the total population were males while 49.51% were females [18]. A substantial area of Sidama land produces coffee, which is the major cash crop in the region, and larger number of the population of the area are known to heavily depend on ‘*Enset*’ *(Enset ventricosum*). The staple foods in Sidama Zone are maize and *kocho* (*false banana)* [19].

In Sidama Zone, there were 18 health institutions, which are providing ART and PMTCT services [20]. Four heath institutions were excluded because they had no eligible study subjects. The characteristics of the four non-selected clusters were compared with the selected clusters and no special characteristics were observed. Hence, the remaining eligible fourteen health institutions were considered as clusters whereby ten health institutions (clusters) were selected randomly.

The sample size was determined using single population proportion formula using a parameter of an expected prevalence for malnutrition among the age group of 6-17 months which was taken as 17.5% [21] with 95% Confidence level, 5% of margin of error. Considering finite population correction formula and adding 20% of the calculated sample for non-responses, the final sample size was 159. Since the calculated sample size (n = 159) is nearly equal to the total number of study subjects in the randomly selected clusters (N = 184), all the eligible mother-child pairs were included and the analysis was based on the 184 study subjects.

### Measurements

Data were collected from mothers as they attended the clinic for follow up. A complementary feeding practice was assessed according to the current recommendation of World Health Organization (WHO). Dietary diversity and meal frequency (i.e indicators of complementary feeding practices) were assessed and determined by single 24 hour dietary recall method [22]. To overcome the limitation of the 24 hour dietary recall method, the study used 7 day quasi food group frequency according to the recommendation of Arimond and Ruel [23]. The food group is based on the current WHO 8 food grouping [22].

### A 24 hour dietary diversity and meal frequency

According to the recommendation of WHO, the optimal number of food groups that a child should consume each day is four or above four. Higher dietary diversity with appropriate meal frequency can help meet daily requirements for a variety of nutrients. The number of food groups and meal frequency consumed in the past 24 hours was assessed by 24 hours dietary recall method.

### Seven-day quasi-food frequency

This was used to overcome the limitation of 24 hours recall method. The same food groups that are used for 24-hours dietary recall were also used in the seven days recall method. This was actually a modified food group frequency, where the questions were asked as “How many days in the last seven days was your child given [food group]? Then, the numbers of days for each food group a child was given was then entered for each child with a maximum of seven. Seven day food group frequency score is created by assigning a score of 0 for each food group if not given in the previous week, +1 if given one to three days, and +2 if given four or more days.

### Data collection procedure

A pre-tested structured questionnaire was used to collect the data. Complementary feeding practice of mothers was assessed by a combination of qualitative 24-hours dietary recall method and seven-day quasi-food frequency. Data collectors were recruited from the respective health institutions and were trained on data collection techniques for 2 days. Data were collected by ten trained nurses and ten assistants from the respective health institutions. Data collectors were closely supervised by the principal investigator. Each filled questionnaire was consistently checked for completeness and consistency.

### Statistical analysis

Before analysis, data were checked for completeness and consistency; the data were entered, cleaned, and coded. Data analysis was carried out by SPSS for windows version 20 (IBM® SPSS® Statistics, IBM Corp, New York). Descriptive statistics was used to show socio demographic characteristics and prevalence of complementary feeding practices. Independent t test, one way ANOVA and binary logistic regression were carried out. To determine the differentials of complementary feeding practices, variables that showed a statistical significant association with complementary feeding practice in the binary logistic analysis were entered into multivariable logistic and multivariable linear regression models accordingly. All tests were two-sided and p value < 0.05 was considered statistically significant.

Ethical clearance was obtained from Hawassa University institutional Review Board (IRB). Letter of permission was also obtained from Sidama zone health department. The objective of the study was explained to the administrative body of the selected health institutions and permission was obtained from the head of the health facilities. Study subjects were assured that the information will be kept confidential and their informed written consent was obtained before interview.

## Result

### Descriptive characteristics

A total of 184 infant mother pairs were included in this study. The mean (±SD) age of mothers was 28.85 (±5.4) years. About fifty five percent (54.9%) of the respondents were protestant by religion and 43.03% were illiterate (do not attended formal education). Above one third (33.7%) of the respondents were Sidama by ethnicity (Table 1).

**Table 1:** **Socio-demographic characteristics of HIV positive mothers, Sidama Zone, Southern Ethiopia, 2012**

***Socio-demographic characteristics***	***Frequency***	***Percentage***
Age of the mother (years)		
≤24	28	15.6
25-29	73	40.8
≥30	78	43.6
Religion (n = 184)		
Protestant	101	54.9
Orthodox	64	34.8
Muslim	15	8.2
Catholic	4	2.2
Ethnic group of mothers		
Sidama	62	33.7
Amhara	37	20.1
Gurage	30	16.3
Oromo	28	15.2
Wolayita	17	9.2
Others*	10	5.4
Marital status		
Married	158	84.5
Widowed	12	6.5
Divorced	11	7.61
Place of residence		
Urban	134	72.8
Rural	50	27.2
Educational status		
Illiterate^ɛ^	77	43.03
Read/write	8	4.47
Primary education (1-8)	53	29.61
Secondary education (9^+^)	41	22.92
Sex of infant		
Male	106	57.6
Female	78	42.4

^ɛ^Those who didn’tattend any formal education *Tigre, Kambata and Gamo.

### Complementary feeding practices

In this study, the prevalence of timely initiation of complementary feeding practice (6-8 months) among HIV exposed infants was 42% [95% CI: (30%-54%)]. The prevalence of timely initiation of complementary feeding practice was not significantly different by place of residence (p = 0.412). There was no statistically significant difference (p = 0.632) between urban and rural HIV exposed infants regarding the age at which complementary feeding was first introduced. The prevalence of bottle-feeding among HIV exposed infant of age 6-17 months was 40.7%. There was statistically significant difference in bottle-feeding practice between urban and rural residents (p = 0.013). The prevalence of minimum dietary diversity was 34.4% and about 53.3% of HIV exposed children did not have the recommended meal frequency for their age in the last 24 recall (Table 2). The mean diet diversity score among bottle fed infants was 2.12 while it was 3.73 among non-bottle fed infants. This difference was statistically significant (p < 0.001). The mean 24-hour meal frequency score among bottle fed infants was 2.15 but the mean 24 hour meal frequency score was 2.79 (p = 0.026) among infants that did not receive bottle feeding. Bottle-feeding had also a statistically significant association with seven-day food group frequency score. Those bottle fed infants had significantly lower food group frequency score (4.11) as compared to their counter parts 6.69 (p < 0.001).

**Table 2:** **Comparison of complementary feeding practices of HIV Positive mothers by place of residence, Sidama Zone, Southern Ethiopia, 2012**

**Feeding practices**		**Urban and rural**	**Urban**	**Rural**	**P-value** ^**¥**^
		**N** **o** **(%)**	**N** **o** **(%)**	**N** **o** **(%)**	
Timely introduction of CF^Ø^	Yes	29 (42.0)	23 (54.0)	6 (31.6)	0.412
	No	40 (58.0)	27 (46.0)	13 (68.4)	
Minimum meal frequency	Yes	84 (46.7)	67 (51.5)	17 (34.0)	0.035
	No	96 (53.3)	63 (48.5)	33 (66.0)	
Minimum dietary diversity	<4	118 (65.6)	80 (61.5)	38 (76.0)	0.067
	≥4	62 (34.4)	50 (38.5)	12 (24.0)	
Bottle feeding	Yes	74 (40.7)	61 (46.7)	13 (74.0)	0.013
	No	108 (59.3)	71 (53.8)	37 (26.0)	
Age at CF started	At 6 months	133 (73.08)	99 (75.6)	34 (68.0)	0.632
	Before 6 months	19 (10.44)	14 (10.7)	5 (10.0)	
	Not started yet	26 (14.29)	16 (12.2)	10 (20.0)	
	don’t know	3 (1.6)	2 (1.5	1 (2.0)	

All calculations were made based on the current WHO indicators for IYCF, ^Ø^Complementary feeding, ^**¥**^Pearson Chi square.

**Figure 1:**
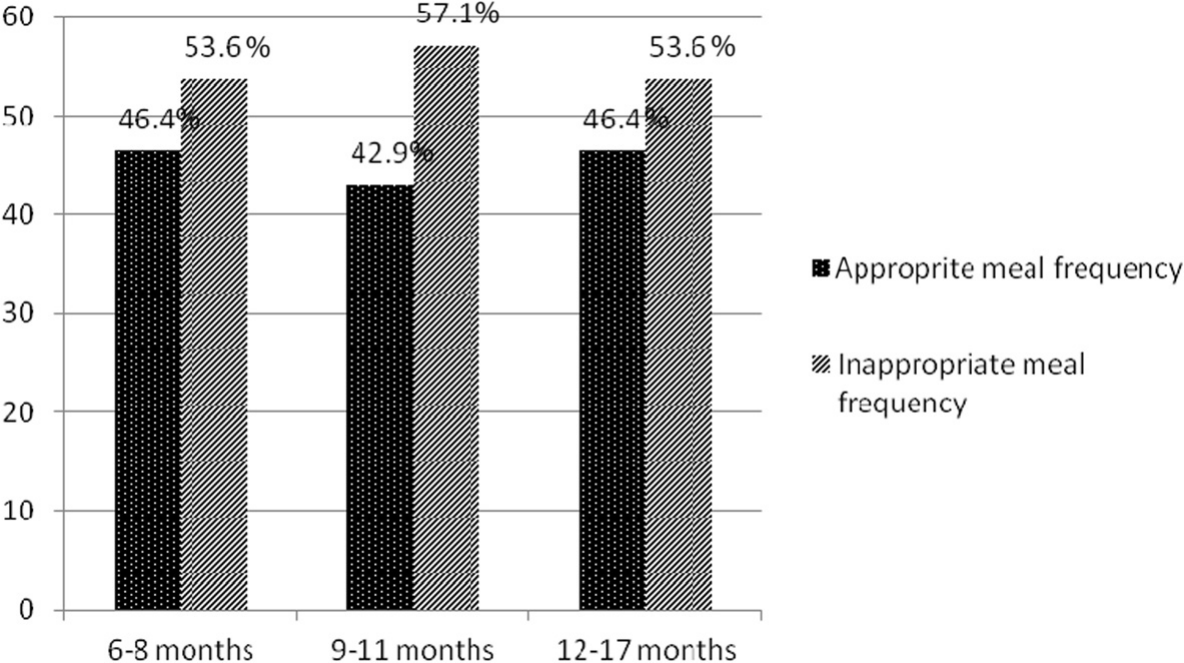
Distribution of HIV exposed infants based on the national age specific IYCF meal frequency recommendation, Sidama Zone, South Ethiopia, 2012.

**Figure 2:**
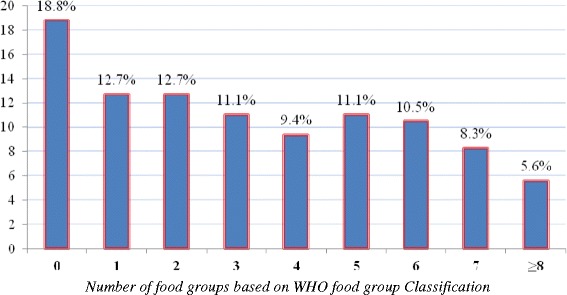
Number of food groups consumed as a complementary food in the last seven days among HIV exposed children, Sidama Zone, Southern Ethiopia 2012.

About 46.4% of HIV exposed infants aged 6-8 months and about 42.9% of infants aged 9-11 months received appropriate(recommended) feeding frequency (Figure 1). The seven-day food group frequency assessment showed that, 18.8% of HIV exposed infants did not consume any of the 8 food groups in the last one week. About 12.7% of HIV exposed infants consumed only one food group while 9.4% of them consumed four food groups in the last one week (Figure 2). In this study, 67% of HIV exposed children consumed whole grains and 22.5% had consumed pulse (plant protein) in the last 24 hours. The consumption of animal sources foods and vitamin A rich fruits and vegetables were 9.9% and 37.4% respectively (Table 3).

**Table 3:** **Food items consumed by HIV exposed infants in Sidama Zone, Southern Ethiopia 2012**

**Food item**		**Frequency**	**Percentage**
Pulses	Yes	41	22.5
	No	141	77.5
Whole grains	Yes	123	67.6
	No	59	32.4
Animal source foods	Yes	18	9.9
	No	164	90.1
Eggs	Yes	57	31.3
	N o	125	68.7
Milk	Yes	82	45.1
	No	100	54.9
Roots and tubers (white potatoes,bulla, kocho, Cassava)	Yes	111	61.0
	No	71	39.0
Vitamin A rich fruit and vegetables.	Yes	68	37.4
	No	114	62.6
Other fruit and vegetables	Yes	41	22.5
	No	141	77.5
Any foods made with oil, fat, or butter	Yes	126	70.0
	No	54	30.0

*N.B. The total number of respondents for each variable is not equal because of missing values.*

There were foods prohibited for infants of age 6-17 months in the study area. The most common prohibited animal source foods were *butter, fatty foods, meat, and egg* while plant source foods such as *banana, kale, pumpkin, avocado, kocho (false banana)* were prohibited foods. About 64.7% of the infants consumed less than four food groups while 35.3% consumed four and above food groups in the last seven days. The seven-day food diversity score showed a strong correlation with 24 hour recall food diversity score (r = 0.97, p <0.001) and seven days food frequency score (r = 0.97, p <0.001).

### Factors associated with complementary feeding practices of HIV exposed infants

There was a statistically significant difference in mean dietary diversity between urban and rural HIV exposed infants. The mean dietary diversity score of infants (based on WHO food groups) was 3.05 (95% CI: 2.61– 3.34) for urban infants and 2.06 (95% CI: 1.43 – 2.64) for the rural infants. The mean meal frequency of food consumed by infants was 2.6 (95% CI: 2.26–2.93) in the urban and 1.98 (95% CI: 1.51-2.48) in the rural area. Similarly, the mean dietary diversity score showed a statistically significant association with ART status (Pre ART vs on ART) of mother (p = 0.048), family size (p = 0.033), number of under five children in the household (p = 0.023), monthly income (p < 0.001) and age of infant (p < 0.001). The mean dietary diversity score had also statistically significant association with presence of food prohibition for infants (p < 0.001) and birth order of the infant (p = 0.017). There was no statistically significant difference in sero status disclosure of the mothers, place of delivery, stigma and discrimination, and ANC attendance of mothers. The meal frequency of HIV exposed infants showed a positive statistical association with infants age (p < 0.001). Meal frequency had a statistically significant association with “time by when mother know her sero status” (p = 0.014) and monthly income of mothers (p = 0.003). Those infants who prohibited from some foods had lower meal frequency as compared to their counter parts (p = 0.008) (Table 4). Multivariable linear regression model showed that age of infants and presence of food prohibition for HIV exposed infants were independent predictors of complementary feeding practices from dietary diversity and meal frequency (Table 5 and Table 6). In multivariable logistic regression analysis education level of the mother had statistically significant association with bottle-feeding practice. HIV positive mothers who had primary education and below were 79% [AOR = 0.21, 95% CI :( 0.06-0.71)] less likely to practice bottle feeding for their infants compared with those with a secondary education and above (Table 7).

**Table 4:** **Factors associated with complementary feeding practices of HIV Positive mothers from 24 hour dietary diversity and meal frequency their infants, Sidama Zone, South Ethiopia, 2012**

**Variables**		**Mean 24 hr DDS** ^**&**^	**Mean 24 hr meal frequency**
Place of residence^α^	Urban	3.05	2.57
	Rural	2.06	1.98
	*P value*	0.007	0.061
ART status of the mother^α^	Pre ART	2.29	2.12
	On ART	2.99	2.54
	*P value*	0.048	0.168
Sero status disclosure of mother^α^	Yes	2.79	2.43
	No	2.65	2.28
	*P value*	0.086	0.72
Stigma and discrimination^α^	Yes	2.94	2.07
	No	2.75	2.44
	P value	0.74	0.47
Presence food prohibited for infants^α^	Yes	2.12	2.15
	No	4.09	2.96
	*P value*	<0.001	0.008
When do you know your sero status*	Before pregnancy	2.82	2.72
	During pregnancy	2.52	1.91
	During birth	1.75	1
	After birth	3.50	2.90
	*P value*	0.25	0.014
ANC^α^	Yes	2.96	2.55
	No	1.79	1.69
	*P value*	0.315	0.075
Place of delivery^α^	Health institution	2.96	2.47
	Home	2.37	230
	*P value*	0.102	0.39
Ag e of the infant*	6-8 months	1.87	1.43
	9-11 months	2.98	2.85
	12-17 months	3.44	3.10
	*P value*	<0.001	<0.001
Birth order*	1	3.13	2.53
	2-3	2.73	2.47
	≥4	2.26	2.00
	*P value*	0.017	0.097
Monthly income (ETB)*^$^	≤500	2.83	2.83
	501-1000	2	1.59
	≥1001	4.10	3.25
	*P value*	<0.001	0.003
Number of under-five children^α^	≤3	2.87	2.53
	>3	1.85	1.65
	*P value*	0.023	0.14
Family size*	≤3	3.38	2.62
	4-5	2.70	2.46
	≥6	2.04	1.86
	*P value*	0.033	0.211

^*α*^ independent t-test, *one way analysis of variance, ^*&*^ *DDS Dietary Diversity Score,* ^$^(Exchange rate 1 USD = 17.87 Ethiopian Birr (ETB)).

**Table 5:** **Multivariable linear regression to identify predictors of 24 hour dietary diversity among HIV exposed infants in Sidama, Zone, South Ethiopia, 2012**

***Variable***	***β***	***P value***
Rural place of residence	-0.158	0.084
ART status (being on ART)	-0.021	0.820
Presence of food prohibition for infants	-0.342	0.001*
Age of the infant	0.311	0.001*
Birth order	-0.202	0.069
Monthly income	0.033	0.723
Number of under five children	0.098	0.291
Family size	-0.150	0.153

*significant at p < 0.05 (two sided)

**Table 6:** **Multivariable linear regression to identify predictors of meal frequency among HIV exposed infants in Sidama Zone, Southern Ethiopia, 2012**

**Variables**	***β***	***P value***
Monthly income (ETB*)	0.042	0.594
Mothers know their HIV status after birth	-0.096	0.200
Age of the child	0.388	<0.001
Presence of food prohibition for infants	-0.181	0.020

*$(Exchange rate 1 USD = 17.87 Ethiopian Birr (ETB)).

**Table 7:** **Predictors of bottle feeding among HIV positive mothers in Sidama Zone South Ethiopia, June 2012**

**Variables**		**COR[95%CI]**	**AOR[95%CI]**
Residence	Urban	2.44 [1.19-5.02]*	1.29 [0.18-9.03]
	Rural	1.0	1.0
Educational status	Primary and below	0.33 [0.14-0.77]*	0.21 [0.06-0.71]*
	Secondary and above	1.0	1.0
Monthly income (ETB)	<500	0.33 [0.11-0.93]*	0.94 [0.22-4.08]
	501-1000	0.38 [0.12-1.24]	0.74 [0.16-3.39]
	>1001	1.0 1.0	
Presence of food prohibition for infant	Yes	4.35 [2.23-8.47]*	1.65 [0.49-5.86]
	No	1.0	1.0

*Significant at p < 0.05(two tailed).

## Discussion

After six months of life, breast milk alone is no longer sufficient to meet the nutritional requirements of infants, and therefore other foods and liquids are needed to complement the breast milk [17]. The quality of children’s diet can have consequences for physical growth, cognitive development, and health. Diet diversity with appropriate meal frequency is recommended to ensure that nutrient needs are met [24,25].

In this study about 42% of HIV exposed infants aged 6–8 months did not receive complementary feeding in the last 24 hours. A comparable finding was reported from Amhara regional state, Ethiopia which showed that 35% of infants aged 6–8 months did not receive complementary foods in the last 24 hours [26]. However, a study from Nepal revealed that 70% of infants aged 6-8 months had been introduced to solid, semi-solid or soft foods [27]. One of the reasons for this difference might result from the difference in socio economic characteristics between the two countries. In our study, there was no statistically significant association between timely initiation of complementary feeding and place of residence which is similar to a study from Bangladesh [28]. A study conducted in Southern regional state, Ethiopia showed that 70.1% HIV positive mothers started complementary food when the child was at 6 months , while about 30% started before 6 months of age which is higher as compared to our findings [29]. This difference might result from the difference in the assessment methodology between the two studies. Our study used the current WHO recommendation for estimating the prevalence of timely initiation for complementary feeding and only infants aged 6-8 months were included [22].

In this study the mean dietary diversity and mean meal frequency were below the WHO recommendation and they were significantly different across the age groups of infants. About two third (65.6%) and 53.3% of the respondents didn’t fulfill the minimum WHO recommendation for food diversity score and meal frequency respectively in the last 24 hours. According to the Ethiopian national nutrition survey report, about 73.7% and 23.7% of children age 6-17 months didn’t fulfill minimum dietary diversity and minimum recommended meal frequency respectively. The current Ethiopian Demographic and Health Survey (EDHS 2011) showed that those infants and young children who started complementary feeding, 48.6% and 4.37% received minimum meal frequency and minimum dietary diversity score respectively [30]. The age specific analysis of meal frequency showed that prevalence of minimum meal frequency to be 46.4%, 42.9% and 46.4% among the age groups 6-8 months, 9-11 months and 12-17 months respectively. This finding is consistent with the EDHS 2011 findings which report that 36.2%, 38.2% and 47.0% among the age groups 6-8 months, 9-11 months and 12-17 months respectively [30]. This findings suggest the need to increase awareness creation activities to the mothers to strengthen the consumption of available food varieties and counselling the mothers at the health facility on food items which ensure the nutrient adequacy of infants.

The age of infants and presence prohibited food for infants were independent predictors of mean dietary diversity of HIV exposed children but, in this study educational status, BMI, attendance of ANC, monthly income and ART status were not statistically associated with complementary feeding practices (meal frequency and dietary diversity). A study in Bangladesh indicated that children of mothers with low BMI, mothers having less antenatal check-ups and rural mothers had significantly lower minimum dietary diversity rate, which are not a statistically significant factors in our findings [28]. A study from Uganda showed that consumption of a diversified diet was significantly associated with socio-economic status [31]. Similarly, a study in Indonesia also showed that mother’s education, rural residence and lower category of infants' age were negatively associated with minimum dietary diversity and minimum meal frequency [32].

In this study, majority (67.6%) of HIV exposed children have complemented with grains in the last 24 hours preceding the survey which is consistent with the current EDHS report [30]. A study conducted in Uganda reported that 88% of households with HIV positive mothers reportedly consumed mainly cereals in the 24 hours prior to the assessment [31]. A study from Bangladesh reported that about 85% of children aged 6–23 months were given grains [28]. The consumption of vitamin A rich fruits and vegetables (37.4%), pulses (22.5%), animal source foods (9.9%), and milk and milk products (45.1%) is consistent with the Ugandan study, which reported that fruits (40.3%) and foods of animal origin like meat (13.9%) and milk/milk products (45.8%) in the last 24 hours. The consumption of legumes was 70% in Uganda [31] while the consumption of pulse in this study was found 22.5%. The justification for the difference may be because Sidama zone is one of the least pulses producing areas in Ethiopia [19].

The Ethiopian EDHS 2011 reported that the prevalence of bottle feeding was 12% [31]. However in this study, 40.7% of HIV exposed children were practicing bottle-feeding in the last 24 hour. The higher prevalence of bottle feeding in this study might resulted from early cessation of breastfeeding due to the fear of mother to child transmission of the HIV virus. Those mothers with lower educational status (primary and below) have a lower chance of bottle feeding as compared to those mothers with educational status of secondary and above. This could be justified by two things; firstly, those mothers with higher educational status were usually employed and were not close to their infant during the working times. So that they start bottle feeding more likely as compared to mothers with lower educational status. Secondly, those mothers with higher educational status might be aware about the mother to child transmission of HIV through breastfeeding. Therefore mothers with higher educational status might believe that switching to bottle feeding from breastfeeding reduce the risk of mother to child transmission of the virus.

One of the limitations of this study is that it used qualitative 24 hour recall which could not able to assess the energy and nutrient intake quantitatively. The other limitation is the recall bias associated with the 24 hour recall and seven day recall.

## Conclusion

About two of every five HIV exposed infants did not timely initiate complementary feeding. The prevalence of bottle feeding practices is high. More than half of the infants did not fulfill the WHO food diversity score and the minimum recommended meal frequency. Higher educational status of the mother was the predictor of bottle feeding while age of infants and presence of food prohibition for HIV exposed infants were independent predictors of 24 hour dietary diversity and meal frequency. Nutritional education on complementary feeding should be designed to improve the feeding practices of HIV exposed children. Increasing awareness activities to the mothers to strengthen the consumption of the available food varieties and items while avoiding bottle feeding.

## References

[1] Jones G, Steketee R, Black R, Bhutta ZA, Morris S, Bellagio/Child/Survival/Study/Group (2003). How many child deaths can weprevent this year?. Lancet.

[2] WHO. Ua: Global Strategy: Breastfeeding critical for child survival. Available at: http://www.who.int/mediacentre/news/releases/2004/pr19/en/(date accessd May 25, 2011) 2004.

[3] WHO/UNAIDS/UNFPA/UNICEF: Guidelines on HIV and infant feeding, Principles and recommendations for infant feeding in the context of HIV and a summary of evidence. Available at: http://whqlibdoc.who.int/publications/2010/9789241599535_eng.pdf (date accessd May 17, 2011). 2010. 24501786

[4] World Bank: HIV/AIDS, nutrition, and food Security: what we can do. A synthesis of international Guidance 2007 pp 30-33. Available at: http://siteresources.worldbank.org/NUTRITION/Resources/2818461100008431337/HIVAIDSNutritionFoodSecuritylowres.pdf (date accessed August 29, 2011). 2007.

[5] WHO: Guidelines for an integrated approach to the nutritional care of HIV-infected children (6 months-14 years) at HIV treatment sites / referral centers. Draft hand book. Geneva, Switzerland. Available at: http://www.unicef.org/aids/files/hiv_6mth-14yr_Handbook.pdf (date accessed December 17, 2012). 2007.

[6] Kebede D, Rett S: Gender, HIV/AIDS and Food Security Linkage and Integration into Development Interventions. DCG Report No. 32. Available at: http://www.drylands-group.org/noop/file.php?id=376. (date accessed at December 17, 2012) 2004.

[7] Alemu A, Bezabih T: The Impacts of HIV/AIDS on Livelihoods and Food Security in Rural Ethiopia: Results from household Survey in Four Regions, World food program(WFP). Available at: http://home.wfp.org/stellent/groups/public/documents/ena/wfp221565.pdf (date accessed at December 25, 2012). 2008.

[8] Vossenaar M, Solomons NW (2012). The concept of “critical nutrient density” in complementary feeding: the demands on the “family foods” for the nutrient adequacy of young Guatemalan children with continued breastfeeding. Am J Clin Nutr.

[9] Dewey K, Brown K (2003). Update on Technical Issues Concerning Complementary Feeding of Young Children in Developing Countries and Implications for Intervention Programs. Food Nutr Bull.

[10] Krebs N, Westcott J (2002). Zinc and Breastfed Infants: If and When Is There a Risk of Deficiency?. Adv Exp Med Biol.

[11] WHO/UNICEF (1998). Complementary Feeding of Young Children in Developing Countries: A Review of Current Scientific Knowledge.

[12] WHO: Complementary feeding: report of the global consultation, and summary of guiding principles for complementary feeding of the breastfed child: Geneva, 10-13 December 2001, Switzerland. Available at: http://www.who.int/nutrition/publications/Complementary_Feeding.pdf (date accessed at December 17, 2012) 2001.

[13] Patel D, Bland R, Coovadiad H, Rollinse N, Coutsoudisd A, Newella M-L (2010). Breastfeeding, HIV status and weights in South African children: a comparison of HIV-exposed and unexposed children. AIDS.

[14] Mugwaneza P, Shema NUW, Ruton H, Rukundo A, Lyambabaje A, Bizimana JD (2011). Under-two child mortality according to maternal HIV status in Rwanda: assessing outcomes within the National PMTCT Program. Pan Afr Med J.

[15] Kimani-Murage EW, Norris SA, Pettifor JM, Tollman SM, Klipstein-Grobusch K, Gómez-Olivé XF (2011). Nutritional status and HIV in rural South African children. BMC Pediatr.

[16] Piwoz E: Nutrition and HIV/AIDS: Evidence, gaps, and priority actions. Available at: [http://reliefweb.int/sites/reliefweb.int/files/resources/BF6867DF2742BEEAC125740F003A9033-fanta_apr2004.pdf] (date accessed January 21, 2013). 2004.

[17] Pan American Health Organization and World Health Organization: Guiding principles for complementary feeding of the breastfed child. December 10-13, 2001. Washington, D.C. 20037. Available at: http://www.who.int/nutrition/publications/guiding_principles_compfeeding_breastfed.pdf

[18] Central Statistics Agency (Ethiopia): Summary and Statistical Report of the 2007 Population and Housing Census, Population Census Commission, Addis Ababa, Federal Democratic Republic of Ethiopia. 2007.

[19] Sidama: an overview of History, culture and economy. Available at: http://sidamachronicle.blogspot.com/2007/11/sidama-overview-of-history-culture-and.html (accessed on September 2012). 2007

[20] Sidama Zone Health Department: Annual reports documents of Sidama zone health department, Hawassa, Ethiopia. 2012.

[21] Ethiopian Health and Nutrition Institute(EHNR): Nutritional baseline survey report for the national nutrition program of Ethiopia: Available at: http://www.ephi.gov.et/images/nutrition/nutrition%20baseline%20survey%20report.pdf (date accessed January 13 2013). 2010.

[22] WHO U, IFPRI, UC Davis, USAID, FANTA, : Indicators for assessing infant and young child feeding practices. Geneva, Switherland World Health Organization (http://www.who.int/maternal_child_adolescent/documents/9789241596664/en/) (date accessd May 25, 2011) 2008.

[23] Arimond M, Ruel M (2002). Summary indicators for infant and child feeding practices: An example from the Ethiopia demographicand health survey 2000.

[24] Crepinsek M, Burstein N: Maternal Employment and Children’s Nutrition Volume I: Diet Quality and the Role of the CACFP. Washington, DC: Economic Research Service; 2004. http://www.ers.usda.gov/publications/efan-electronic-publications-from-the-food-assistance-nutrition-research-program/efan04006-1.aspx. In*.*; 2004.

[25] Women Infants and Children Program: Infant feeding guidelines and pamphlets. Available at: http://www.cdph.ca.gov/programs/wicworks/Pages/WICNEInfantFeedingGuidelinesandPamphlets.aspx. 2007.

[26] Nekatebeb H, Guyon A, Beyero M, Stoecker BJ: Factors related to exclusive breast feeding and dietary diversity of complementary foods: A case study in Amhara region of Ethiopia. Journal of federation of American societies for experimental biology. Abstract; 2010, 24 (734).

[27] Joshi N, Agho K, Dibley M, Senarath U, Tiwari K (2006). Determinants of inappropriate complementary feeding practices in young children in Nepal: secondary data analysis of Demographic and Health Survey. Matern Child Nutr.

[28] Kabir I, Khanam M, Agho KE, Mihrshahi S, Dibley MJ, Roy SK (2012). Determinants of inappropriate complementary feeding practices in infant and young children in Bangladesh: secondary data analysis of Demographic Health Survey 2007. Matern Child Nutr.

[29] Feleke B (2010). Assessment of child feeding practice among HIV positive mothers In selected Southern Ethiopia PMTCT and ART clinics.

[30] Central Statistical Agency (CSA) Ethiopia: Demographic and Health Survey 2011. Addis Ababa, Ethiopia and Calverton, Maryland, USA: CSA and ORC Macro. 2011.

[31] Bukusuba J, Kikafunda J, Whitehead R (2009). Nutritional status of children (6-59 months) among HIV-positive mothers/caregivers living in an urban setting of Uganda. Afr J Food, Agric, Nutr Dev.

[32] Ng C, Dibley M, Agho K (2012). Complementary feeding indicators and determinants of poor feeding practices in Indonesia: a secondary analysis of 2007 Demographic and Health Survey data. Public Health Nutr.

